# Comparative analysis of the effects of collection methods on salivary steroids

**DOI:** 10.1186/s12903-021-01722-w

**Published:** 2021-07-16

**Authors:** Ce Zhu, Chao Yuan, Qidi Ren, Fangqiao Wei, Shunlan Yu, Xiangyu Sun, Shuguo Zheng

**Affiliations:** 1 Department of Preventive Dentistry, Peking University School and Hospital of Stomatology, National Center of Stomatology, National Clinical Research Center for Oral Diseases, National Engineering Laboratory for Digital and Material Technology of Stomatology, Beijing, 100081 People’s Republic of China; 2 Department of Preventive Dentistry, Shanghai Ninth People’s Hospital, Shanghai Jiao Tong University School of Dentistry, Shanghai, 200011 People’s Republic of China

**Keywords:** Salivary steroids, Flow rate, Concentration, Secretion rate, Comparative analysis

## Abstract

**Background:**

Steroid hormone test for saliva was a promising area of research, however the impact of different collection methods on salivary steroids was underexplored so far. This study was designed to compare the effects of different collection methods (unstimulated or stimulated by chewing paraffin, forepart or midstream) on salivary flow rate, concentrations and secretion rates of steroids in saliva.

**Methods:**

Whole-saliva samples were collected from 10 systemically and orally healthy participants, whose forepart and midstream segments of saliva were collected under unstimulated and stimulated conditions, with the salivary flow rate of each sample recorded. The concentrations and secretion rates of salivary steroids including testosterone, dehydroepiandrosterone (DHEA) and progesterone were measured by ELISA, with the multiple of change calculated.

**Results:**

The results indicated mechanical stimulation used in collection of saliva samples could affect concentrations and secretion rates of steroids, whereas forepart and midstream segments had little differences in levels of salivary steroids, which effects could be partly influenced by individual specificity. The asynchronism in change of secretion rate of steroids with that of salivary flow rate might play an important role during this course.

**Conclusion:**

Based on these findings, we suggested to use the same collection method throughout one analytical study on salivary steroids or in longitudinal observations to ensure the comparability of the saliva samples collected.

**Supplementary Information:**

The online version contains supplementary material available at 10.1186/s12903-021-01722-w.

## Background

Recently, salivary biomarkers were unveiled to be significantly associated with oral and systemic diseases [1–3], with a steady stream of new research utilizing salivary biomarkers to evaluate disease risk, to identify oral and systemic diseases at an early stage, to monitor potential response to treatment, and to better introduce and discuss the role of antibiotics prescriptions against biofilm as well as on salivary biomarkers inflammation (e.g. Galectin, suPAR, IL-6, etc.) [4–8]. Salivary steroids had become one kind of the most widely studied biomarker, with previous literature demonstrated that the levels of steroids in saliva could reflect the corresponding conditions in blood [9, 10].

Saliva, as an emerging pool of biomarkers, could be collected via non-invasive, painless, simple and convenient procedures with minimal biological risks [1, 11]. The ultrafiltration form of saliva could harbour free hormones with biological activities, indicating that salivary steroids test could be more sensitive for assessing the fraction of steroids which were biologically functional to the target tissues [9, 12, 13]. Besides, the sampling procedures of saliva could be repeated for several times within the same day, which made duplicate analyses for monitoring the levels of steroids much easier, offering a path to present the secretion rhythm precisely and control the dose of hormones effectively [14, 15]. In this context, the utilization of saliva for monitoring the level of steroids had attracted growing attention in medical research and clinical applications in the recent few years.

Saliva could be secreted by three major salivary glands (parotid, submandibular and sublingual glands) and more than 100 minor glands. However, for sample collection on a patient basis, mixed whole saliva was verified as the sole option which was practically feasible in clinical circumstances [16, 17]. Some researchers believed that salivary steroids were not affected by external stimulation as they derived from blood by passive diffusion in the unbound form [9, 18]. However, this problem might be more complicated than they expected as some other studies demonstrated that chewing gum could interfere with the assessment of certain categories of steroids, but there were also conflicting results from different studies on the same steroids [19–23]. Thus, the effects of collection methods (unstimulated *versus* stimulated) on levels of salivary steroids remained controversial at present.

As reported by previous research on steroids in urine, the midstream urine sample was much cleaner and more stable than the forepart, hence should be used as the golden standard of sampling for urinalysis [24]. Nevertheless, whether saliva had similar properties in different segments was still questionable without sufficient evidence, though several previous studies had already request participants to discard or swallow the forepart of saliva and keep the midstream segment for subsequent analysis [25–27].

Based on the above, collection methods might affect the composition of saliva samples, including salivary steroids which had certain potentiality to be used as biomarkers for assessing healthy status or discovering disease at an early stage. Hence, the aim of this study was to perform comparative analyses of the effects of different collection methods (unstimulated or stimulated, forepart or midstream) on salivary flow rate, concentrations and secretion rates of steroids in saliva, directing the way to use these collection methods in future analytical studies on salivary steroids.

## Methods

### Study population

In the present study, 10 participants were recruited in accordance with the following inclusion criteria: (1) ≥ 18 years old; (2) no history of systematic diseases; (3) no history of antibiotic therapy within the last 3 months; (4) not pregnant or lactating currently; (5) no smoking and alcohol-drinking habit; (6) no presence of untreated dental caries, periodontal diseases, or other oral diseases. The exclusion criteria for the present study were: (1) patients with xerostomia or hyposalivation; (2) poor compliance with the study procedures; (3) refusal to sign the informed consent.

This study followed the STROBE guideline checklist, with its ethical approval obtained from the Peking University School and Hospital of Stomatology Ethics Committee (PKUSSIRB-201944061). Two dental clinicians were involved in the present study, who performed review of medical history and oral clinical examination, respectively. All the participants had signed the informed consent before the study commenced.

### Sampling and processing of saliva

Participants were requested not to eat, tooth brushing, drink, exercise, or chew gum for at least 2 h, and were instructed to rinse their mouth thoroughly with water at about 10 min before the sampling started [28]. Saliva collection procedures were carried out at 9:00–10:00 a.m. With the assistance of a funnel (ZhenQi, Shanghai, China), whole-saliva samples were collected into a 5-mL polypropylene tube (cat. no.:0030108302, Eppendorf, Hamburg, Germany) which had the 1.5 mL scale.

**Fig. 1:**
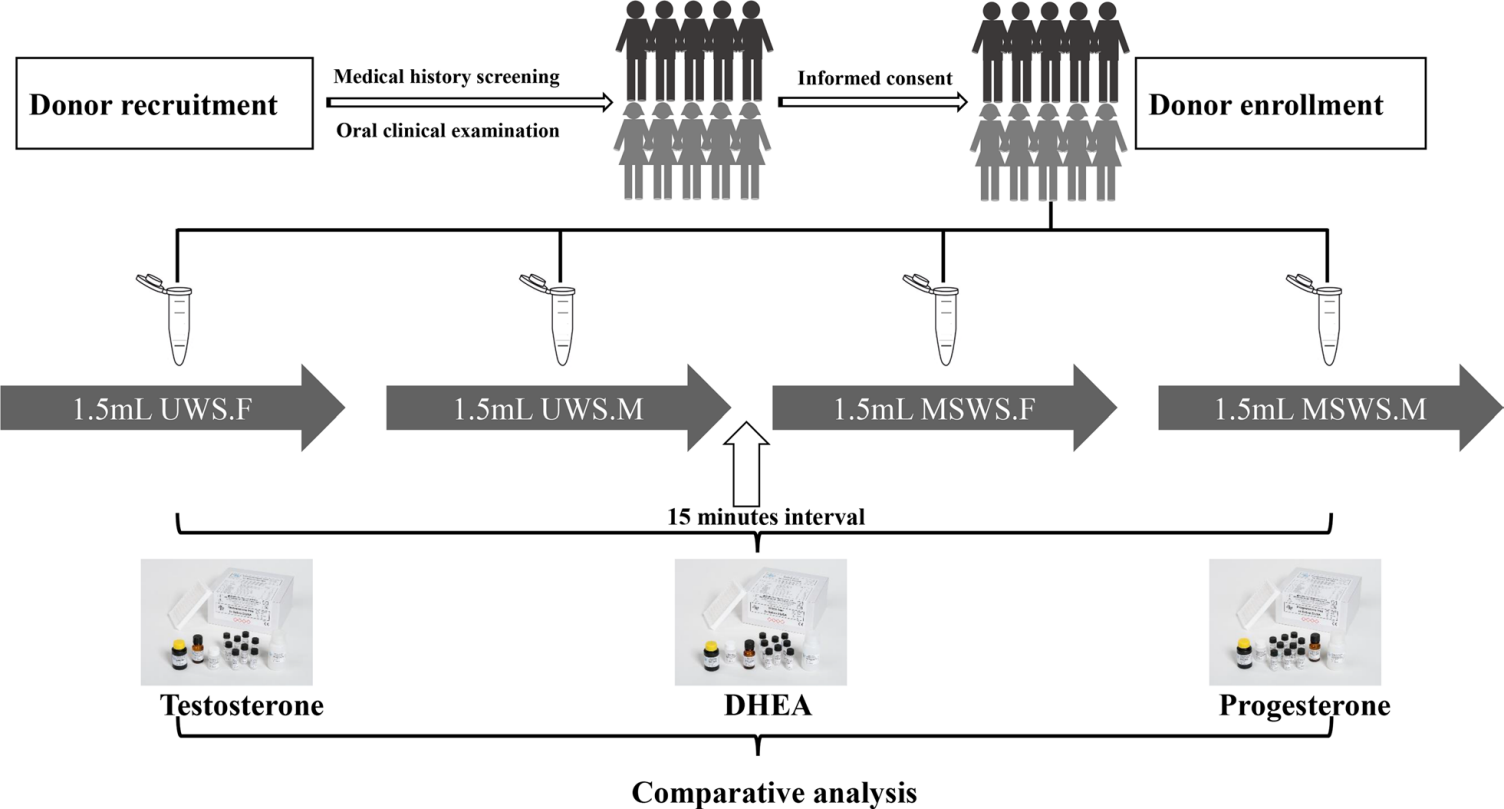
The flow chart of this study, exhibiting the flow path from the recruitment of participants to comparative analysis. UWS.F, the forepart segment of unstimulated whole saliva. UWS.M, the midstream segment of unstimulated whole saliva. MSWS.F, the forepart segment of mechanically stimulated whole saliva. MSWS.M, the midstream segment of mechanically stimulated whole saliva (similarly hereinafter)

The four collection methods and sequence were as follows (Fig. 1): Initially, 1.5 mL unstimulated whole saliva (forepart, marked as UWS.F) was collected into one tube by passive drooling, followed immediately by 1.5 mL unstimulated whole saliva (midstream, marked as UWS.M) using another collecting tube. Then, after a 15-min interval for rest, 1.5 mL whole saliva with mechanical stimulation (forepart, marked as MSWS.F) by chewing a prescriptive paraffin gum (with a frequency of once per second to keep the constant sialogogue effect) was collected into a new collecting tube, after which 1.5 mL stimulated whole saliva with the same stimulation (midstream, marked as MSWS.M) was collected into another tube. For each tube (sample), the volume of saliva was measured by the scales on the tube (~ 1.5 mL), and the flow rate was then calculated by dividing its measured volume by the time duration of sample collection.

Saliva samples were immediately placed on ice, transported to the laboratory within 2 h, and then centrifuged at 10,000×g for 10 min at 4 °C. For each sample, the supernatant was transferred to a new separate tube and stored at − 80 °C for further use.

### Measurement of concentrations of steroids in saliva

Blood contamination in saliva samples was detected using the salivary transferrin/blood contamination ELISA kit (Salimetrics, Carlsbad, USA) [29, 30], and those samples with a transferrin level of greater than 0.5 mg/dL were excluded according to the instructions.

After thawed at 4 °C, the saliva samples were used for assays to measure concentrations of three free steroids [testosterone, dehydroepiandrosterone (DHEA) and progesterone] using the commercialized ELISA kits (Demeditec Diagnostics, Kiel-Wellsee, Germany) according to the manufacturer’s instructions. All the experimental operations were carried out by the same researcher, and all the analyses were performed in duplicate. In the present study, the four-parameter logistic curve fitting coefficients of the calibration curve for testosterone, DHEA and progesterone were 0.995, 0.999 and 0.996, respectively (Additional file 1: Figure S1). The extent of dilution effect was calculated to preliminarily learn the influence of mechanical stimulation on levels of steroids in saliva.

### Secretion rates of steroids and the multiple of change

Secretion rate was calculated by multiplying the concentration of designated steroid with the flow rate measured during saliva collection. For more comprehensive comparisons, we also employed a specific value called the multiple of change, which was calculated by the following formula: The multiple of change = $\frac{{{\text{secretion rate }}\left( {{\text{stimulated condition}}} \right)}}{{{\text{secretion rate}}\left( {{\text{unstimulated condition}}} \right)}}$. Values of “the multiple of change” were then compared with the ratio of flow rate [$\frac{{{\text{flow rate }}\left( {{\text{stimulated condition}}} \right)}}{{{\text{flow rate }}\left( {{\text{unstimulated condition}}} \right)}}$] (as control) to assess the effects of mechanical chewing stimulation on secretion process of steroids.

### Statistical analysis

Data analysis was performed using SPSS 23.0 software (IBM, Armonk, NY, USA). One-way repeated measures analysis of variance (ANOVA) was employed to compare the measurement indicators (flow rate, concentration and secretion rate of steroids) between different collection methods, with a post-hoc Bonferroni correction used for multiple comparisons. The following comparisons were reported: UWS.F vs MSWS.F, UWS.M vs MSWS.M, UWS.F vs UWS.M, and MSWS.F vs MSWS.M.

The details of one-way repeated measures ANOVA were listed as follows: If *P* (Mauchly's test of sphericity) ≥ 0.05, the *P* values of sphericity assumed were adopted; if *P* (Mauchly's test of sphericity) < 0.05, and the epsilon (ε) < 0.75, *P* values of Greenhouse–Geisser were adopted; if *P* (Mauchly's test of sphericity) < 0.05, and the epsilon (ε) > 0.75, *P* values of Huynh–Feldt were adopted. *P* < 0.05 was regarded as statistical significance (two-sided).

## Results

All the 40 saliva samples had passed the blood contamination test and were eligible for the subsequent comparative analyses among saliva samples collected with different methods (UWS.F, UWS.M, MSWS.F, and MSWS.M).

### Comparative analysis of salivary flow rate

**Fig. 2:**
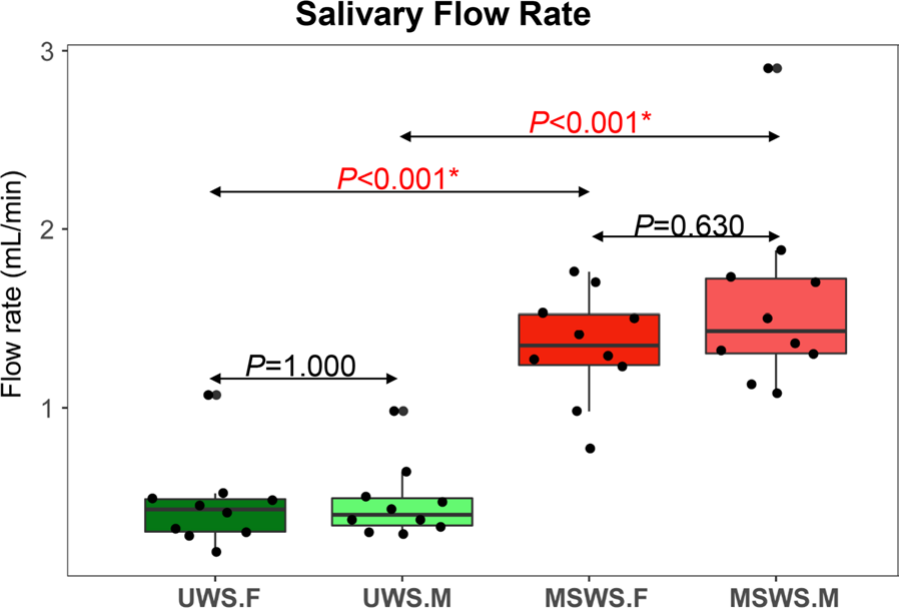
Comparative analysis of salivary flow rates between different groups (UWS.F, UWS.M, MSWS.F and MSWS.M, similarly hereinafter). *P < 0.05

The mean flow rate for UWS.F was 0.45 ± 0.24 mL/min, while that for UWS.M was 0.47 ± 0.21 mL/min. MSWS.F exhibited a mean flow rate of 1.34 ± 0.31 mL/min, while this parameter for MSWS.M was 1.59 ± 0.53 mL/min. As shown in Fig. 2, a significant difference was found as expected in the flow rates between unstimulated and mechanically stimulated saliva in both the forepart and midstream segments (*P* < 0.001). No statistical significance was observed in comparison of flow rates between different segments of saliva in spite of stimulation (*P* = 1.000 for unstimulated condition, *P* = 0.630 for stimulated condition, respectively).

### Comparative analysis of concentrations of steroids in saliva

**Fig. 3:**
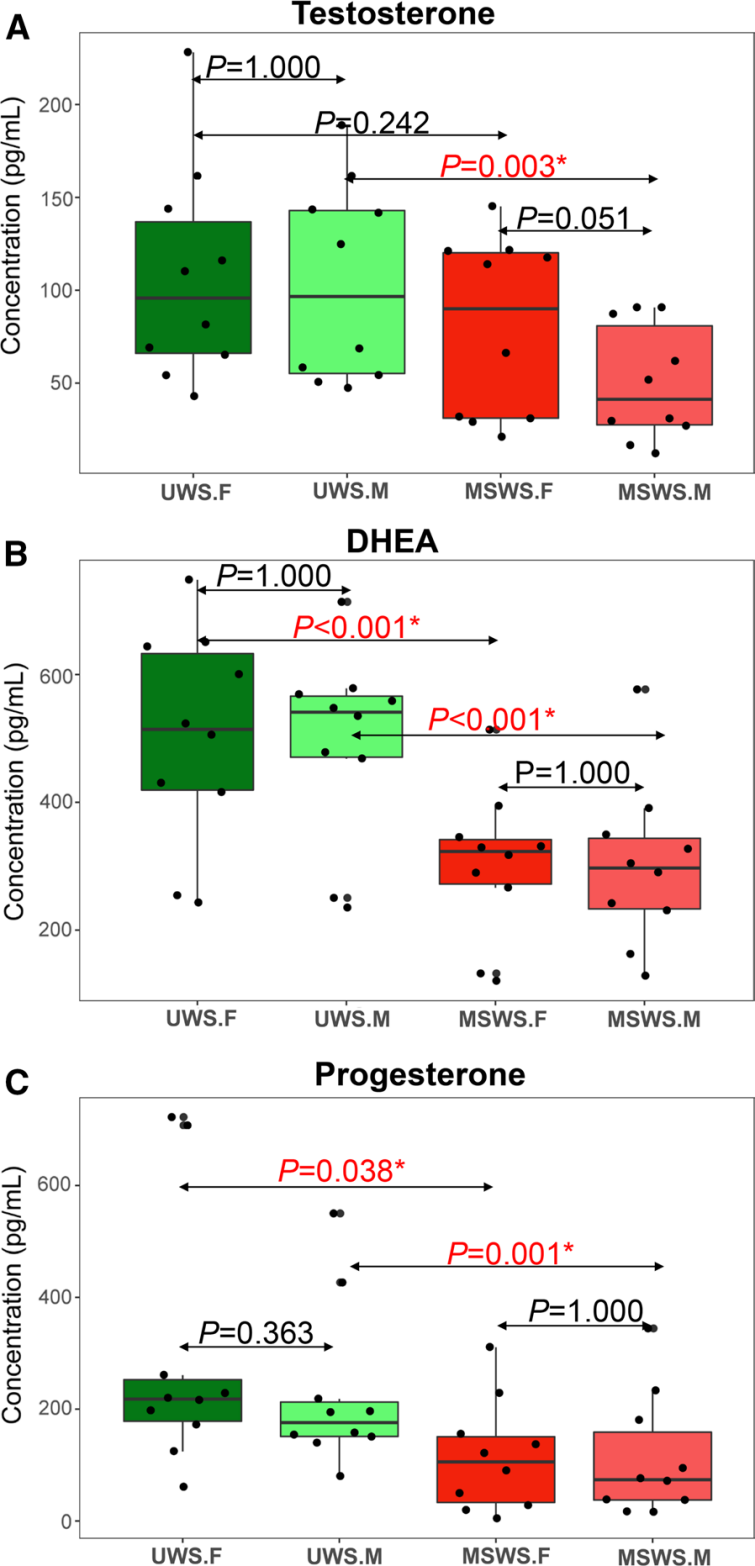
Comparative analysis of concentrations of the three designated steroids between different groups (**a** for testosterone, **b** for DHEA, and **c** for progesterone). Horizontal lines represent the mean and standard deviation (SD) values. *P < 0.05

The three kinds of salivary steroids (testosterone, DHEA, and progesterone) exhibited relatively similar patterns in comparison of concentration of steroids between saliva samples collected with different methods. As shown in Fig. 3, the concentrations of all the three kinds of steroids in UWS.M group were significantly higher than those in MSWS.M group (*P* = 0.003 for testosterone, *P* < 0.001 for DHEA, and *P* = 0.001 for progesterone, respectively), while UWS.F group had significantly higher level of concentrations of DHEA and progesterone than MSWS.F group ((*P* < 0.001 for DHEA, *P* = 0.038 for progesterone, respectively). There were no significant differences in other comparisons (*P* > 0.05).

For the forepart segment, the extent of dilution effect were 27.4% ± 28.0% for testosterone, 40.1 ± 8.5% for DHEA, and 57.3% ± 29.7% for progesterone, respectively. Meanwhile, for the midstream segment, the extent became 53.4% ± 15.7% for testosterone, 40.0 ± 11.0% for DHEA, and 56.8% ± 24.5% for progesterone, respectively. These results indicated that mechanical stimulation by chewing paraffin could result in attenuation of concentrations of salivary steroids when compared with unstimulated condition. However, no consistent tendency was found in comparison of the extent between different segments (forepart and midstream) of saliva.

### Comparative analysis of secretion rates of steroids and the multiple of change

**Fig. 4:**
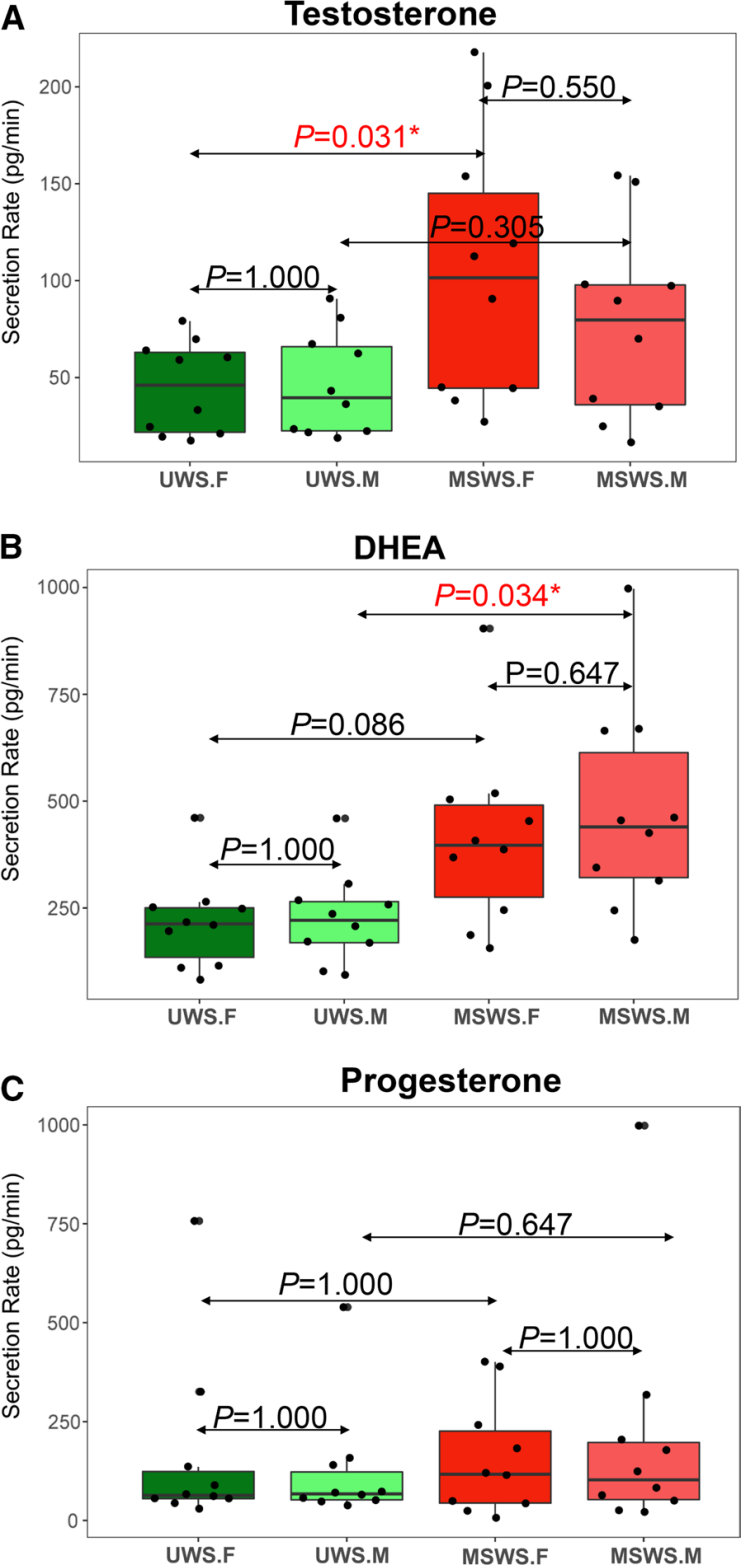
Comparative analysis of secretion rates of the three designated steroids between different groups (**a** for testosterone, **b** for DHEA, and **c** for progesterone). *P < 0.05

**Fig. 5:**
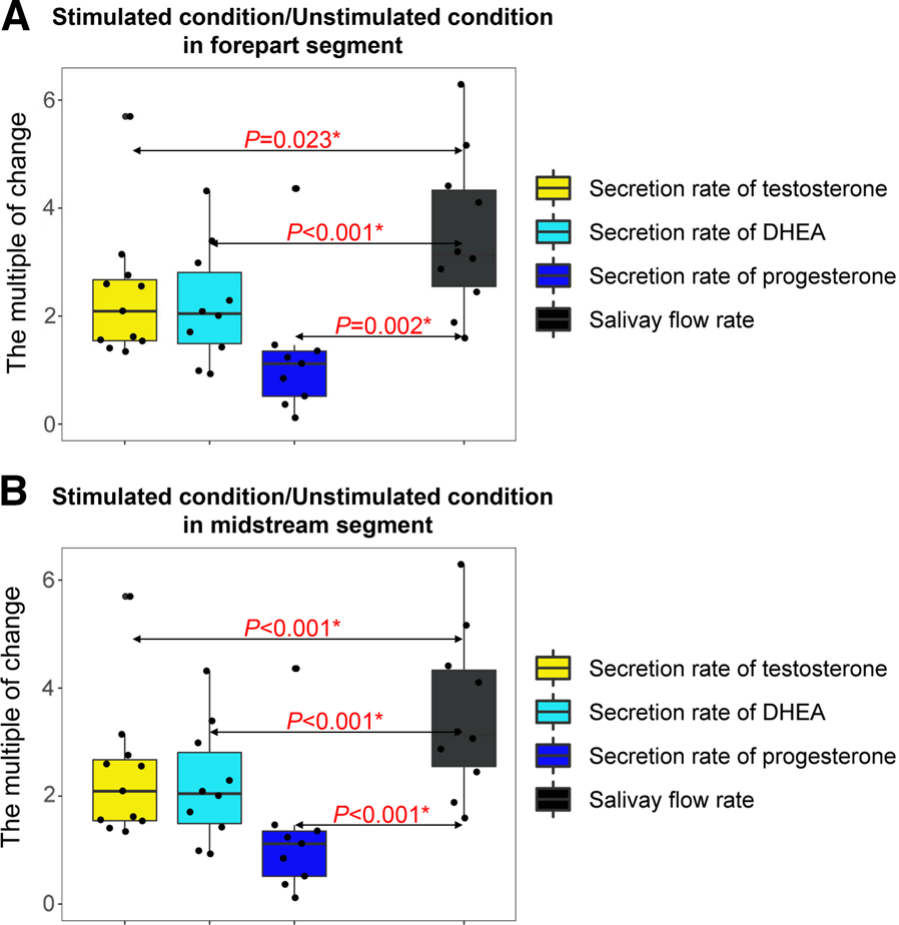
Comparative analysis of the multiple of change of the three designated steroids and the ratio of flow rate (**a** for forepart segment, **b** for midstream segment). *P < 0.05

Figure 4 demonstrated the variations of secretion rate of steroids in saliva samples collected by different methods. For testosterone, secretion rates in UWS.F group were significantly lower than those in MSWS.F group (*P* = 0.031); while for DHEA, UWS.M group had significantly lower secretion rates than MSWS.M group (*P* = 0.034). Other comparisons exhibited no statistical differences. Further investigation on the multiple of change (Fig. 5) showed the increment of secretion rates of the three steroids were significantly less than that of salivary flow rate (*P* < 0.05), implying that the attenuation of concentrations of salivary steroids in response to mechanical stimulation might be caused by the asynchronism in change of secretion rate of steroids with that of salivary flow rate.

## Discussion

With a series of comparative analyses, the present study confirmed that salivary flow rate, concentrations and secretion rates of steroids in saliva and the multiple of change would exhibit a variation in response to mechanical stimulation during collection of saliva samples but had little differences between forepart and midstream segments. These findings opened a window for analytical research on salivary steroids with regards to the use of different collection methods.

In the past few decades, saliva had attracted more attention as an alternative body fluid to serum or plasma, as it contained a large number of biomarkers which was closely associated with certain systemic and oral diseases, e.g. periodontal infection diseases [4–6]. Salivary steroids, as a hotspot category of biomarkers in saliva, had become one advantageous option to assess the hormone level associated with a variety of fields including psychiatry, stress research, endocrinology, and sports medicine [9, 31, 32]. Although saliva had not yet become a mainstream sample source for hormone analysis, it was proved to be reliable and, in some cases, even superior to other body fluids [9, 12, 13, 33]. But there was still a long way to go for salivary tests to be accepted by clinicians due to the lack of specific and standardized collection methods to achieve comparable salivary hormone data. There were conflicting results on whether and how collection methods (unstimulated or stimulated, forepart or midstream segments) affected the levels of steroids in saliva based on literature review. In this context, the present study aimed to compare the effects of these collection methods on the levels of salivary steroids, which was, to our knowledge, the first study to carry out the comparative analysis of the effects of collection methods both on the concentrations and secretion rates of steroids in saliva.

Three steroids, namely testosterone, DHEA and progesterone, were selected into the present study based on the following evidence: (1) the concentration of these three hormones in saliva was relatively high; (2) these three hormones were useful indicators in numerous clinical and health-related diagnoses[12, 18, 34, 35]; 3) Their diurnal rhythms were relatively stable in the morning [9, 10, 12]. In the present study, all the free steroid commercialized ELISA kits for saliva were qualified with acceptable quality control sample ranges. Moreover, the fitting coefficients of the calibration curves were fairly high (Additional file 1: Figure S1). The concentration ranges of salivary steroids in our study were consistent with previous studies reported [36–39], and so was their range of coefficient of variation (0.11–0.31, logarithmically transformed for comparisons) [28, 40, 41], indicating that the measurement of steroids in the present study was reliable.

It was reported in the literature that the mean salivary flow rate was 0.1–0.5 mL/min at rest; however, stimulation by chewing paraffin or other ways could increase this rate by 2–5 times [42]. The results of salivary flow rate in this study confirmed this point as well. According to previous literature reported, submandibular and sublingual secretory cells were most responsible for the resting secretion, whereas the parotid provided the major contribution to secretion in response to external stimulation [43]. Thus, it was speculated that mechanical stimulation by chewing paraffin or other ways would change not only the flow rate of saliva secretion, but also the main source of glands for secretion.

Hormones could penetrate from blood circulation into saliva by a variety of mechanisms, which speed was controlled by the lipophilic layers of the capillaries and glandular epithelial cells [9, 44]. Some researchers believed that salivary steroids concentrations are unaffected by collection methods as they are transported from blood to saliva as the unbound form by passive diffusion [9, 18]. Interestingly, there were conflicting findings in the effects of unstimulated and stimulated conditions on the concentrations of salivary steroids based on a review of previous studies. Schultheiss [20] demonstrated that mechanical chewing could reduce the concentration of salivary testosterone, whereas Büttler et al. [19] and Anders [21] had got the inverse conclusion. For cortisol, Büttler et al. [19] found chewing activity could raise its concentration in saliva, but Schultheiss [20] observed the opposite trend. In the present study, we observed effects of mechanical stimulation on decreased concentrations and increased secretion rates of certain designated steroids, providing evidence that changes in saliva flow rate and main source of salivary glands had great contribution to secretion of these salivary steroids.

The issue brought by forepart and midstream segment in saliva sampling was not given due attention by previous studies, with some researchers preferred to preserve midstream saliva by discarding or swallowing the forepart segment [25–27]. However, little was known so far on whether the secretion rhythms and main source of salivary glands had changed between the forepart and midstream segments. In the present study which was the first study to investigate this problem, no significant difference of salivary flow rates was observed between different segments in spite of mechanical stimulation, whereas concentrations of salivary steroids were minimally affected by segments. These findings refreshed our knowledge on the segment effect of salivary steroids and confirmed that discarding or swallowing the forepart segment was not necessary for collection of saliva samples in analytical research on steroids.

Our results on secretion rates of salivary steroids indicated that attenuated concentrations of salivary steroids were caused by the relatively lower rate of hormone secretion compared with changes in the salivary flow, which mechanism still required more in-depth research to be explored in further. For comparisons of secretion rates of steroids in midstream segment between unstimulated and stimulated conditions, similar phenomenon (comparatively low level in UWS.M, but higher in MSWS.M) was observed for all the three kinds of steroids, but only DHEA exhibited statistical significance (*P* < 0.05, Fig. 4), revealing potentially distinct secretion characteristics of different kinds of steroids in saliva. In consideration of limited sample size in the present study, we were not able to arbitrarily conclude that mechanical chewing stimulation could significantly increase the secretion of certain steroids like DHEA, but the trend shown in the figure might imply that variations in salivary steroid levels did exist if different segments or stimulation methods were used for collection of saliva samples, enlightening us about the effects of different sampling methods on levels of biomarkers such as steroids.

Inter-individual variation might be an probable factor that affected the patterns of steroids as reported previously [28]. Research conducted by Stone et al. [45] revealed that some people do not have the typical diurnal rhythm of salivary cortisol secretion, whereas Hansen et al. [46] confirmed that changing rhythm of salivary hormones in the morning exhibit two distinct trends. The contradiction of whether and how collection methods (unstimulated *versus* stimulated) affect the steroid level in previous studies might be introduced by the inter-individual variation. Given the limited sample size of our study, we conducted a sensitivity analysis to explore the influence of individual specificity by removing each participant in turn to investigate if the results would alter due to the change of study population. As shown in Additional file 1: Table S1, for the comparison between different segments of mechanically stimulated saliva, the removal of Participant 01 or 10 would lead to a statistical significance. On the other hand, for the comparison of forepart segment between unstimulated and stimulated conditions, the removal of Participant 02, 04, 06, 07, 08, or 09 would also give rise to the disappearance of statistical significance. All the other statistics were not changed if one of the participants was removed. This sensitivity analysis revealed that individual specificity would slightly affect the concentrations of steroids in saliva, implying the necessity to validate the present findings in a larger population.

Importantly, findings of the present study must be interpreted within the context of certain study limitations. First, our main findings were all based on ten healthy subjects with both males and females involved, resulting in the necessity to be cautious when making any further extrapolation. Although the effects of gender differences were not suitable for a statistical comparison due to the limited sample size, the influences of different gender on salivary steroids should be taken into account at the study-designing stage in all up-coming research in the future. Second, the results would be more convincing if the cross-over study design was used to reduce the influences of different sample-collection sequence, though we have designed a 15-min resting interval before the change of stimulation method to enable a recovery of saliva gland function and alleviate the potential effects of sampling sequence. Future studies with cross-over study design and longitudinal investigation of dynamic changes could greatly improve the quality of research to yield more valuable results in this field. Third, the volume of saliva was measured by the scales on the tube, which accuracy still had some room for improvement though we mainly focused on the overall concentrations of steroids in the present study. More accurate measurements would be much helpful to strengthen the estimate of concentrations and secretion rates of steroids in saliva, particularly for future studies in a larger population.

## Conclusion

In conclusion, mechanical stimulation by chewing paraffin used in collection of saliva samples could affect concentrations and secretion rates of three typical steroids (testosterone, DHEA, and progesterone) in saliva, whereas forepart and midstream segments had little differences in levels of salivary steroids, which effects could be partly influenced by individual specificity. The asynchronism in change of secretion rate of steroids with that of salivary flow rate might play an important role during this course. Therefore, it was suggested to use the same collection method (unstimulated or stimulated, forepart or midstream) throughout one analytical study on salivary steroids or in longitudinal observations to ensure the comparability of the saliva samples collected.

## Supplementary Information

**Additional file 1**. Calibration curves (**Figure S1**) and sensitivity analysis (**Table S1**)

## Data Availability

All data generated or analysed during this study are included in this published article and the Additional file 1.

## Abbreviations

suPAR: Soluble urokinase plasminogen activator receptor
IL-6: Interleukin-6
STROBE: Strengthening the reporting of observational studies in epidemiology
PKUSSIRB: Peking University School (and Hospital) of Stomatology Institutional Review Board
UWS.F: Forepart segment of unstimulated whole saliva
UWS.M: Midstream segment of unstimulated whole saliva
MSWS.F: Forepart segment of mechanically stimulated whole saliva
MSWS.M: Midstream segment of mechanically stimulated whole saliva
ELISA: Enzyme linked immunosorbent assay
USA: United States of America
DHEA: Dehydroepiandrosterone
SPSS: Statistical product and service solutions
IBM: International business machines corporation
NY: New York
ANOVA: Analysis of variance
