# Supplementary material for: Comparative analysis of the effects of collection methods on salivary steroids
Source: BMC Oral Health. 2021 Jul 16;21:352. doi: 10.1186/s12903-021-01722-w (PMC8285872; doi:10.1186/s12903-021-01722-w)
Supplement: Supplementary file 1 — Additional file 1. Calibration curves (Figure S1) and sensitivity analysis (Table S1) [file 12903_2021_1722_MOESM1_ESM.docx]

Supplementary file for

**Comparative analysis of the effects of collection methods on salivary steroids**

by Ce Zhu, Chao Yuan, Qidi Ren, Fangqiao Wei, Shunlan Yu, Xiangyu Sun, and Shuguo Zheng

**
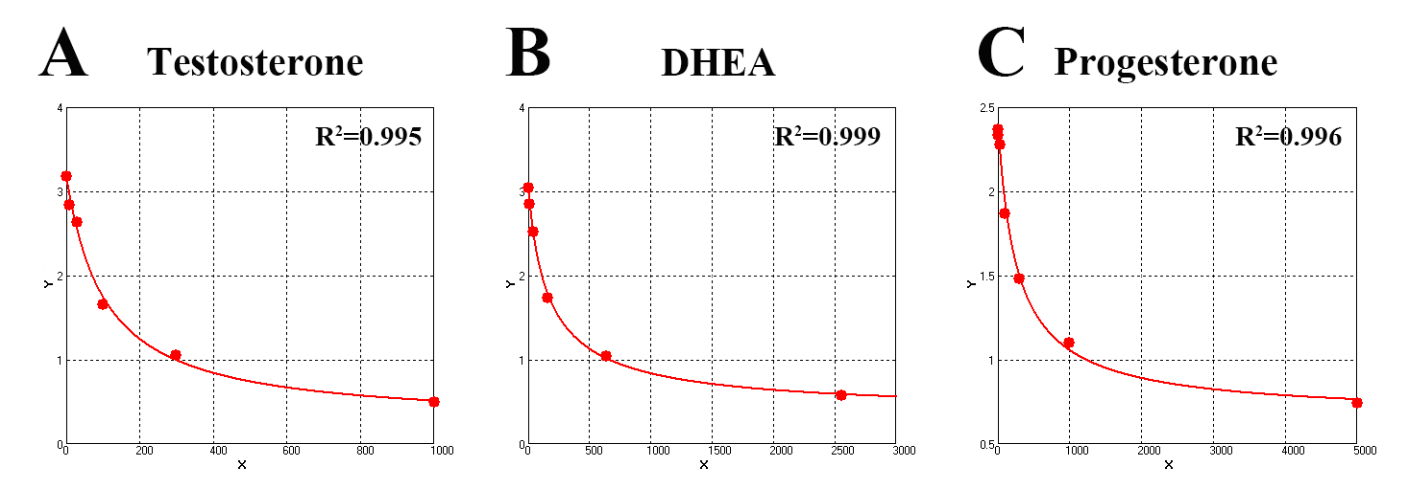
Figure S1.** Calibration curves showed the fitting coefficients of free steroids in saliva ELISA kits.

**Table S1**. Sensitivity analysis on concentrations of steroids in saliva by removing each participant in turn.

|  |  |  |  |  |  |  |  |  |  |  |  |  |
| --- | --- | --- | --- | --- | --- | --- | --- | --- | --- | --- | --- | --- |
| Participant ID | Testosterone | | | | DHEA | | | | Progesterone | | | |
|  | *P*_UWS.F-UWS.M_ | *P*_MSWS.F-MSWS.M_ | *P*_UWS.F-MSWS.F_ | *P*_UWS.M-MSWS.M_ | *P*_UWS.F-UWS.M_ | *P*_MSWS.F-MSWS.M_ | *P*_UWS.F-MSWS.F_ | *P*_UWS.M-MSWS.M_ | *P*_UWS.F-UWS.M_ | *P*_MSWS.F-MSWS.M_ | *P*_UWS.F-MSWS.F_ | *P*_UWS.M-MSWS.M_ |
| All samples | 1.000 | 0.051 | 0.242 | 0.003 | 1.000 | 1.000 | <0.001 | <0.001 | 0.363 | 1.000 | 0.038 | 0.001 |
| 01 | 1.000 | 0.035^#^ | 0.327 | 0.004 | 1.000 | 1.000 | <0.001 | <0.001 | 0.311 | 1.000 | 0.031 | 0.001 |
| 02 | 1.000 | 0.125 | 0.204 | 0.008 | 1.000 | 1.000 | <0.001 | <0.001 | 0.485 | 1.000 | 0.079^#^ | 0.001 |
| 03 | 1.000 | 0.097 | 0.382 | 0.008 | 1.000 | 1.000 | <0.001 | <0.001 | 0.531 | 1.000 | 0.048 | 0.003 |
| 04 | 1.000 | 0.104 | 0.169 | 0.005 | 1.000 | 1.000 | <0.001 | <0.001 | 0.460 | 1.000 | 0.081^#^ | 0.004 |
| 05 | 1.000 | 0.096 | 0.196 | 0.005 | 1.000 | 1.000 | <0.001 | <0.001 | 0.487 | 1.000 | 0.048 | 0.003 |
| 06 | 1.000 | 0.111 | 0.227 | 0.006 | 1.000 | 1.000 | <0.001 | <0.001 | 0.747 | 1.000 | 0.052^#^ | 0.003 |
| 07 | 1.000 | 0.074 | 0.361 | 0.006 | 1.000 | 1.000 | <0.001 | <0.001 | 0.422 | 1.000 | 0.078^#^ | 0.004 |
| 08 | 1.000 | 0.060 | 0.358 | 0.006 | 1.000 | 1.000 | <0.001 | <0.001 | 0.331 | 1.000 | 0.080^#^ | 0.003 |
| 09 | 1.000 | 0.050 | 0.490 | 0.005 | 1.000 | 1.000 | <0.001 | <0.001 | 0.429 | 1.000 | 0.086^#^ | 0.004 |
| 10 | 1.000 | 0.041^#^ | 0.449 | 0.003 | 1.000 | 1.000 | <0.001 | <0.001 | 0.299 | 1.000 | 0.040 | 0.002 |

Participant ID: The individual who was removed in turn. ^#^: Indicating change of statistical significance.

UWS.F, the forepart segment of unstimulated whole saliva. UWS.M, the midstream segment of unstimulated whole saliva. MSWS.F, the forepart segment of mechanically stimulated whole saliva. MSWS.M, the midstream segment of mechanically stimulated whole saliva.
